# English Medicine, Medical Books and Authors—American Books in England—Journals, Etc.

**Published:** 1874-09-01

**Authors:** 


### ENGLISH MEDICINE, MEDICAL BOOKS AND AUTHORS—AMERICAN BOOKS IN ENGLAND—JOURNALS, ETC.

*London Correspondence in the Clinic, August 1st*, 1874.

WHATEVER may be said of the work done by the physicians of other nationalities, it must be admitted, I think, that the place filled by the contributions of English medicine is one of the largest and most important. Billroth, a typical German, and, as everybody knows, one of the foremost medical men of our time, pays a just tribute to English physicians when he declares in his introduction to his Surgical Pathology, that the most important contributions to our science have been made in England. The conservatism, the cautious habit of the English mind, and, I may add, its honesty, have, it is true, apparently hindered the development of English medicine, but have certainly established on a firmer basis all the improvements in our science and art coming from English sources.

The English medical authors, as I have intimated in previous letters, are chiefly the younger men, who alone have the time to undertake original investigations or to engage in the labor of literary composition. The book, whether intended to represent practical or scientific medicine, whether a compilation of existing knowledge on the subject treated of, or intended to put forth the results of experimental research, is usually a venture made by the author himself with the object of improving his position in the profession and of introducing him into practice. There are but few London medical men who devote themselves exclusively to scientific medicine, and the most of the really satisfactory work in this direction is accomplished under great disadvantages by those who are struggling into practice.

Most of the English medical works are pecuniary ventures of their authors, and no risks are assumed by the publishers. I was informed by Dr. Beale that he personally superintended every stage in the publication of his works, selecting paper and type and witnessing the making and printing of the illustrations. The sale of the book, if successful, reimburses the author for his expenditure, but the chief recompense comes from the increased business which the book brings. Not unfrequently a book on some special disease or group of diseases, is put forth merely as an advertisement. One may see in the secular press, especially in the *Times*, advertisements of these works with commendatory notices annexed. This mode of bringing themselves before the public, employed, too, by reputable men, has, however, been recently sharply rebuked by the medical journals, and has been officially inquired into and condemned by some of the societies.

Whilst it is true that the physicians of the United States have been so largely dependent on English sources for their supplies of medical information, it is now quite apparent that a small but increasing current of medical literature is setting in from the United States to England.

The medical journals of London are very powerful and influential. The number of weeklies is a clear indication of the intellectual activity of the medical profession. There are three great weeklies — the *Lancet*, *British Medical Journal*, and the *Medical Times aad Gazette* — all representatives of British medical opinion, but preserving individual peculiarities and appealing to different influences in the profession for support. The *Lancet* has the largest circulation, especially amongst lay readers, and is to be found in all of the club houses, public libraries and in many private houses. The old animosities which were excited by the *Lancet* at its foundation and for a few years subsequently, have entirely disappeared. The paper is owned by the Wakleys, the two sons of its founder. With success it has become conservative, but is still independent. It is edited, not by the Wakleys, the owners, but by young men, able, sprightly and rising writers, employed by them for this work. As a consequence of this system, the editors are frequently changed, but the policy of the paper remains the same. The *Lancet* has become quite a valuable property and nets, it is said, five thousand pounds per annum.

The *British Medical Journal* is the organ of the British Medical Association, and has the support of that powerful body. This journal has probably the largest circulation in the profession. It is very ably edited by Mr. Earnest Hart. Besides conducting the *British Medical*, Mr. Hart edits two other weekly journals, *The Medical Record* and *The Sanitary Record;* the first named being made up chiefly of abstracts of important papers published in foreign journals, and the last named being devoted to subjects in sanitary science. It would be quite impossible for one man to perform this enormous labor unless he possessed the facility of Mr. Hart in this kind of work, and relinquished all other engagements except editorial as Mr. Hart does.

The *Medical Times and Gazette* has a much smaller circulation than the other great weeklies, but it is a journal of very lofty tone and represents the more conservative elements in English medical politics. It has been a long time edited by Dr. Druitt, the well-known author of the text book on surgery. Ill-health lately compelled Dr. Druitt to seek relief in the climate of Madras, and during his absence the journal has been extremely well conducted by Dr. Cholmoley. I have heard that Dr. Druitt has recently returned, much improved in health, and that he will again undertake the editorial management of the journal.

There is another very lively little monthly journal published in London entitled *The Doctor*. It is owned and edited by Chapman, the spinal ice-bag man. It is very independent, rather saucy, and represents the opinions of a few *guerillas*, who are at war against the existing medical status. Chapman is also owner and editor of the *Westminster Review*, a quarterly journal which represents whatever is most radical in English politics, morals and religion. Beside the editorial charge of the periodicals, Chapman is a general practitioner, using his spinal ice-bags, chiefly, I believe, in the treatment of disease.

Besides the weeklies, there are two quarterly medical periodicals, *The British and Foreign Medico-Chirurgical Review* and *The Journal of Mental Science*, and a monthly, *The Practitioner*, edited by Dr. Anstie. The patronage extended to so many journals published in one city, certainly justifies the remark that it indicates a high degree of intellectual activity. The elevated tone of these journals, their keen regard for the interests of the medical profession, and their hearty condemnation of whatever is low and unworthy in the conduct of medical men, demonstrate their fitness for the important position which they assume as representatives of English medicine.

---

DELIRIUM TREMENS.—M. Magnan, in a communication to the *Societe de Biologie* (quoted by Dr. R. Lepine in the, *Gazette Med. de Paris*, 1873, No. 22), gives the following thermometric characters of the grave and mild forms of *delirium tremens:*

In the milder form the temperature hardly exceeds 38° cent. (100.4 F.) It oscillates about this figure, but does not notably exceed the normal temperature. If, therefore, the temperature of the patient during the first two or three days does not exceed 38.5°, it may be taken for granted that he will not succumb to that attack. The progress of the temperature is altogether different in the grave type of the disease. It rapidly reaches 39°, oscillating between that figure and one more elevated for two or three days, then rising to an ultimate height of 40 or 41° (104, 105.8° F.). Death occurs soon after the appearance of this increased temperature. It is also in this form that is observed another symptom, insisted upon by M. Magnan—a sort of general tremor of all the muscular fibres.

M. Magnan has also called attention, in his lectures at Ste. Anne, to the hemi-anæsthesia which is sometimes observed in chronic alcoholism, and of which he had observed seven cases within a few years.

In one case well marked, the force was much diminished in the limbs of the right side and the sensibility was entirely lost, there being not merely anæsthesia of the skin, but also loss of the muscular and tactile sense, the sight, smell, and taste: only the hearing remained on that side and that is much impaired.

M. Magnan, principally from the observations of Turck, believes the lesion which produces so remarkable a hemi-anæsthesia, can be localized in the optic thalamus, though its exact nature, owing to the want of autopsies, remains at present unknown.

M. Magnan's article at length in *Gaz. Med. de Paris*, No. 24, June 14, 1873.

